# Is secondhand smoke exposure associated with depressive symptoms among secondary school students in Malaysia? Findings from a national school-based study

**DOI:** 10.18332/tid/197278

**Published:** 2025-03-18

**Authors:** Kuang Hock Lim, Yoon Ling Cheong, Chee Cheong Kee, Sumarni Mohd Ghazali, Mohd Hazilas Mat Hashim, Ali Aman Marine, Jia Hui Lim, Hui Li Lim

**Affiliations:** 1 Institute for Medical Research, National Institutes of Health, Ministry of Health Malaysia, Kuala Lumpur, Malaysia; 2 Biostatistics and Data Repository Sector, National Institutes of Health, Ministry of Health Malaysia, Shah Alam, Malaysia; 3 Institute for Medical Research, National Institutes of Health, Ministry of Health Malaysia, Shah Alam, Malaysia; 4 School of Pharmacy, Faculty of Health and Medical Sciences, Taylor's University, Subang Jaya, Malaysia; 5 Institute for Clinical Research, National Institutes of Health, Ministry of Health Malaysia, Shah Alam, Malaysia

**Keywords:** secondhand smoke, depression symptom, NHMS 2017: AHS, school-going adolescents

## Abstract

**INTRODUCTION:**

Numerous studies have shown that secondhand smoke (SHS) is harmful to human health. Thus, the purpose of this study was to look into the relationship between exposure to SHS and depression among secondary-school students in Malaysia.

**METHODS:**

We derived the data from the Malaysian National Health and Morbidity Survey 2017: Adolescents Health Survey (NHMS 2017: AHS). We examined the association between SHS exposure and depression in 24497 secondary school students. Descriptive and multivariable logistic regression analyses were performed.

**RESULTS:**

The study revealed that 42% of the students were exposed to SHS during the last seven days. Depression symptoms were associated with SHS exposure (AOR=1.16; 95% CI: 1.07–1.25) after adjusting for possible confounding effects of other independent variables, including age, gender, ethnicity, smoking status of respondents, marital status of parents, physically being bullied, and physical and verbal abuse.

**CONCLUSIONS:**

To prevent and control school-going adolescents’ exposure to SHS, health education and smoking cessation among those who have close contact with adolescents should be enhanced. In addition, promoting more smoke-free areas, including houses and public places, should be intensified among secondary school students in Malaysia as they transition to adulthood.

## INTRODUCTION

Secondhand smoke (SHS) consists of both exhaled mainstream smoke, which is the smoke breathed out by the smoker, and sidestream smoke, which comes from the burning tip of a cigarette^1^. Exposure to SHS increases the risk of diseases and dying from cardiovascular conditions such as coronary heart disease, lung cancer, chronic obstructive pulmonary disease, and other types of cancer^2^, and is especially detrimental to the respiratory development of children^3^. Children are more susceptible to the harmful health effects of SHS exposure because of their underdeveloped immune systems, faster breathing rates, and smaller size, which lead them to absorb a more significant amount of pollutants^4^. This is evidenced by mortality due to SHS contributing to 37 million disability-adjusted life years (DALYs), with 11.2% of the burden in children aged <5 years^5^. Many studies have revealed that SHS contains various harmful substances, including nicotine, carbon dioxide, carbon monoxide, carbonyl compounds, hydrocarbons, polycyclic aromatic hydrocarbons, nitrosamines, and tar^6^. Among those chemicals, nicotine is the primary psychoactive component of nicotinic acetylcholine receptors, which are involved in acetylcholine^7^. Numerous clinical and preclinical studies have shown that nicotine impacts mood, aggression, and related concepts and behaviors, like agitation and irritability^7^. The function of nicotinic acetylcholine receptors in regulating mood and anxiety has been clarified. Exposure to tobacco smoke has acute and long-term effects on the dopamine system, which may lead to long-term imbalances in dopamine transport^8^. Lower levels of dopamine result from prolonged exposure to tobacco smoke and have also been related to an increased risk of negative mood or depression^9^. Another biological mechanism that refers to tobacco smoke and SHS exposure to depressive symptoms is chronic inflammation^10^.

Consequently, SHS has an association with neuropsychiatric illnesses and mental health issues^11^, apart from health problems such as cancer and CVD^2^. SHS can increase the risk of depression via stress, chronic adverse physical conditions, and the biological effects of nicotine^11^. Indeed, several studies conducted among adults have found that SHS increases the risk of depressive symptom onset^12,13^; the same findings were also reported among youth and adolescents, either in the local study, national school, or systematic review of the findings of a survey of the association between SHS exposure and depression/depression symptoms in developed and developing countries. All the studies showed a significant positive association and higher odds of depression/depression symptoms among adolescents who were exposed to SHS^14-16^. The findings are worrisome, given that globally more than 6 in 10 adolescents (62.9%) are exposed to SHS, and for South-East Asia it is higher (65.3%)^17^.

Similarly, Malaysia is facing a similar situation, considering that various studies at the national and large-scale levels in this country have found that 42–58% of secondary school adolescents are exposed to SHS^18,19^. In addition, The National Health and Morbidity Survey: Adolescent Health Survey (NHMS 2022: AHS) 2022 found that more than a quarter (26.9%) of secondary school adolescents in Malaysia reported depressive symptoms^20^; while the Singh et al.^21^ study among 1350 adolescents aged 13–14 years across nine secondary schools in Selangor state reported that the prevalence of depressive symptoms among all participants was 19% (95% CI: 16.9–21.2), with a higher prevalence of depressive symptoms being reported among females 26.3% (95% CI: 23.0–29.8) compared to males 11.7% (95% CI: 9.4–14.4).

It has been reported that the developing brain in children and adolescents is particularly susceptible to substances found in SHS (e.g. nicotine)^22^. In addition, developing knowledge and skills, learning to manage emotions and relationships, and acquiring attributes and abilities, also apply during adolescence. Additionally, the adolescent years are critical for an individual’s health in adulthood because they are the time when they undergo a variety of physical, social, and psychological changes and are exposed to health risk factors. Adolescent behavior is likely to be carried into adulthood^23^, and the same is true of other health conditions, such as mental health, where studies reveal that the majority of adolescents (75%) who have major depressive disorder will experience depression in adulthood.

The effect of SHS calls for proactive measures. To ensure the effectiveness of interventions by stakeholders (e.g. Ministry of Health, Malaysia) to reduce SHS exposure among adolescents in Malaysia, local evidence must be obtained since adaptation or adaptation from other countries may have a different efficacy than evidence in this country^24^. In addition, with proof of the health effects on their children, parents and guardians may be more encouraged to implement a policy or intervention, and allowing policymakers to enact appropriate policies and measures to reduce exposure to SHS among adolescents^25^. This study aims to investigate the effect of exposure to SHS and its association with depression/depression symptoms among secondary school students in Malaysia.

## METHODS

### Study design

We derived the data from the National Health and Morbidity Survey 2017: Adolescent Health Survey (NHMS 2017: AHS). The study employed a cross-sectional and multistage sampling method to select a representative sample of secondary school students aged 13–17 years in Malaysia. The first sampling stage was the stratification of states in Malaysia, followed by the stratification of each state based on the urban and rural areas. The Ministry of Education and Rural and Regional Development provided the sampling frame based on enrolment in 2016. The primary sampling unit was the selection of schools from each state by systematic random sampling (212 secondary schools), and the secondary sampling unit was the selection of classes from each selected school (4–10 classes). Proportionate size sampling was employed to select the class from each school. We invited all students from the school chosen to participate in the study. The sample size was based on the variance of interest (3%), margin of error (3%), and confidence interval of 95%; the design effect of two was obtained from the 2012 GSHS-Malaysia study, anticipating a 25% non-response rate. The sample size required was 30496 respondents.

### Measures

We employed the active consent approach to obtain data from the selected respondents. A letter describing the details of the study, such as the study’s objective, the respondents’ anonymity, and the consent form, was sent to the parents/guardians through the school administration. Those parents/guardians who consented to their child participating in the study returned the consent form – only those respondents who returned the consent form were allowed to participate. The data were collected in the school’s specific areas without teachers/staff from the schools before the surveyed session. The trained research assistants were briefed on the study’s objective, that participation was voluntary, the information given would only be used for research purposes, and they were briefed on the items in the questionnaire. The research team members assisted the respondents who needed help to understand the questionnaire’s specific items. The Malaysia Ministry of Education approved the protocol of the study, and the Medical Research and Ethical Committee, Ministry of Health, Malaysia, granted the ethical approval for the study (NMRR-16-698-30042).

The questionnaire was adopted from the Malaysia Global School Health Surveyed 2012, which had been validated^20^. The dependent variable (depression) was examined using the validation Malay version of the DASS 21 questionnaire. For seven items measuring depression, it was shown to have high reliability among Malaysian Youth (Cronbach’s alpha 0.917)^26^, the respondents were required to select the condition appropriate to them in the past week (e.g. ‘I found it challenging to work up the initiative to have done things’, with the choice of never=0, sometimes=1, often=2, and almost always=3). The score obtained was multiplied by 2. Respondents who scored 0–9 were classified as having no depression, while those who scored ≥10 were categorized as having depressive symptoms^27^.

The independent variables were gender, ethnicity, study level [upper secondary (16–17 years) or lower secondary (aged 13–15 years)], marital status of parent/guardian, if they were attacked physically at least once (yes/no), had at least one episode of physical abuse (yes/no), were subject to verbal abuse (yes/no), bullied at least once (yes/no), and if they had a close friend (yes/no). Adolescents that smoked at least once during the lasted 30 days were categorized as current smokers, and respondents reported being exposed to someone smoking in front of them at least one day in the previous seven days, were classified as being exposed to SHS.

### Data management and analysis

The data were cleaned and checked for inconsistencies. The sample weight was calculated to adjust the sample data to ensure sample representativeness in line with the complex sampling analysis method. The weight used for the sample weight estimation was: (W = W1 × W2 × F × PW), where W1 is the inverse of the probability of selecting the school, W2 is the inverse of the probability of choosing the classroom within the school, F is the inverse of the response rate of school level, class, and student, and PW is the post-stratification adjustment factor based on gender and class of the students.

We used descriptive statistics to describe the characteristics of respondents. The chi-squared analysis was used to determine the association between SHS and other independent variables. We included the factors of the bivariate analysis with a p≤0.25 in the multivariable logistic regression model. The effect size was reported as an odds ratio and 95% CI. Two-way interaction was performed between SHS exposure and the other independent variables (gender, class of study, marital status of respondent’s parent, smoking status, verbally abused at least once, physically abused at least once, physically attacked at least once, and bullied at least once). We ran the statistical analysis at an alpha value of 0.05. We used SPSS statistical software version 22 for all statistical analyses.

## RESULTS

The response rates for NHMS 2017: AHS was 89% (n=27497/30496), the equal gender distribution was observed among the sample (male 49.6%), approximately 6 in 10 respondents were aged 13–15 years and of Malay ethnicity (Table 1). Almost all (96%) of the respondents reported they had a close friend, and almost 9 out of 10 had parents who were married. More than 40% reported having been verbally abused and more than one-tenth of respondents (11.8%) reported having been physically abused at least once. A subset of 42% of respondents reported being exposed to SHS at least once during the last week.

**Table 1:** Sociodemographic characteristics of secondary school students who participated in the NHMS 2017: AHS (N=27497)

*Characteristics*	Estimated* population	*n*	*%*	*95% CI*
**Gender**				
Male	1064953	13135	49.6	46.5–52.7
Female	1081492	14362	50.4	47.3–53.5
**Ethnicity**				
Malay	1354538	18713	63.1	57.7–68.2
Chinese	358504	4100	16.7	13.4–20.6
Indian	149227	1428	7.0	5.2–9.3
Bumiputra Sabah	149354	1781	7.0	4.3–11.1
Bumiputra Sarawak	96823	924	4.5	2.6–7.6
Other	37997	554	1.8	1.3–2.5
**Age** (years)				
13–15	1302499	16952	60.7	56.8–64.5
16–17	843946	10545	39.3	35.6–43.2
**Marital status of parents**				
Married	1834475	23544	87.4	86.7–88.0
Divorce/separated	264521	3387	12.6	12.0–13.3
**Physical attack at least once**				
Yes	542467	6735	25.3	24.1–26.6
No	1600544	20722	74.7	73.4–75.9
**Physical abuse at least once**				
Yes	253449	3034	11.8	10.8–12.9
No	1888965	24416	88.2	87.1–89.2
**Bullied at least one**				
Yes	346882	4436	16.2	15.2–17.2
No	1795518	23022	83.8	82.8–84.8
**Verbal abuse at least once**				
Yes	924222	11681	43.2	41.8–44.7
No	1214101	15727	56.8	55.3–58.2
**Having a close friend**				
Yes	2058223	25424	96.4	96.8–95.9
No	77103	942	3.6	3.2–4.1
**Current smoker**				
Yes	342209	4140	15.9	14.7–17.3
No	1803548	22437	84.1	82.7–85.3
**Exposed to SHS**				
Yes	900560	11385	42.0	40.5–43.6
No	1242418	16070	58.0	56.4–59.5

* The sample was weighted based on the probability of selecting the school, classroom response rate of school level, class, and student, and post-stratification adjustment on gender and form of the students.

The prevalence rate of depressive symptoms was 32.8%, in which the prevalence was significantly higher among respondents of Indian descent (47.2 %), those being verbally (42.3%) and physically abused (54.3%), being physically attacked (44.5%) and bullied at least once (52.7%). The study also found that 45.4% of smokers reported depression symptoms. Similarly, school students who reported being exposed to SHS at least once during the last week also showed a higher proportion of having depression symptoms compared to their counterparts who were not exposed to SHS in the previous seven days (36.0% vs 30.4%, p<0.001) (Table 2).

**Table 2:** Association of sociodemographic variables, being victimized, smoking status, and SHS exposure with depression symptoms among secondary school students in Malaysia among participants in the NHMS 2017: AHS (N=27497)

*Variable*	*Depression symptoms*								
	*Yes*				*No*				
	*Estimated population	*n*	*%*	*95% CI*	*Estimated population	*n*	*%*	*95% CI*	*p*
**Gender**									
Male	333015	4010	32.2	30.6–33.8	701615	8783	67.8	66.2–69.4	0.233
Female	352643	4615	33.4	32.0–34.8	703833	9437	66.6	65.2–68.0	
**Ethnicity**									
Malay	395731	5547	30.0	28.7–31.3	923856	12731	70.0	68.7–71.3	<0.001
Chinese	124375	1378	35.3	32.8–38.6	227530	2647	64.7	62.0–67.2	
Indian	67888	597	47.2	42.2–52.2	75944	787	52.8	47.8–57.8	
Bumiputra Sabah	56203	637	38.8	35.0–42.8	88548	1089	61.2	57.2–65.0	
Bumiputra Sarawak	28286	269	29.9	26.3–33.8	66317	633	70.1	66.2–73.7	
Other	13180	197	36.2	29.7–43.2	23251	333	63.8	56.8–70.3	
**Education level**									
Lower secondary	412135	5317	32.5	31.1–34.0	855645	11237	67.5	66.0–68.9	0.557
Upper secondary	273524	3308	33.2	31.4–35.1	549803	6983	66.8	64.9–68.6	
**Marital status of parents**									
Married	564903	7142	31.6	30.5–32.7	1223229	15871	68.4	67.3–69.5	<0.001
Divorce/separated	99149	1222	38.6	36.2–41.1	157445	2057	61.4	58.9–63.8	
**Physical attack at least once**									
Yes	234551	2856	44.5	42.5–46.6	292105	3700	55.5	53.4–57.5	<0.001
No	449947	5752	28.8	27.7–29.9	1111662	14502	71.2	70.1–72.3	
**Physical abuse at least once**									
Yes	133484	1594	54.3	51.6–57.0	112153	1347	45.7	43.0–48.0	<0.001
No	550250	7009	29.9	28.8–31.0	1291568	16852	70.1	69.0–71.2	
**Bullied at least one**									
Yes	176318	2213	52.7	50.2–55.2	158514	2088	47.3	44.8–49.8	<0.001
No	506808	6391	28.9	27.9–30.0	1245696	16117	71.1	70.0–72.1	
**Verbal abuse at least once**									
Yes	380703	4788	42.3	40.7–43.9	519523	6612	57.7	56.1–59.3	<0.001
No	301959	3802	25.5	24.3–26.8	881282	11561	74.5	73.2–75.7	
**Having a close friend**									
Yes	38399	479	51.3	46.6–56.5	36120	433	48.5	43.5–53.4	<0.001
No	643301	8095	32.1	31.0–33.2	1363026	17718	67.9	66.8–69.0	
**Current smoker**									
Yes	98852	7436	45.4	43.5-50.5	118757	1497	54.6	51.2–57.9	<0.001
No	584745	1160	31.3	30.2-32.4	1283585	16679	68.7	67.6–69.8	
**Exposed to SHS**									
Yes	315753	3889	36.0	34.4–37.6	561332	7218	64.0	62.4–65.6	<0.001
No	368566	4721	30.4	29.2–31.7	842456	10981	69.6	68.3–70.8	

* The sample was weighted based on the probability of selecting the school, classroom response rate of school level, class, and student, and poststratification adjustment on gender and form of the students.

Supplementary file Figure 1 presents two-way interactions between SHS exposure and the other independent variables (gender, class of study, marital status of respondent’s parent, ethnicity, smoking status, being verbally abused at least once, being physically abused at least once, being physically attacked at least once, and being bullied at least once), but no significant two-way interactions were found.

Multivariable logistic regression analysis showed that the variables, which showed significant associations with depression/depression symptoms in the bivariate analysis, were also crucial in multivariate analysis. Respondents who were exposed to SHS showed 16% higher odds of being depressed/having depressive symptoms compared to their counterparts after adjusting for the confounding effect of other independent variables (Table 3).

**Table 3:** Multivariable logistic regression analysis to determine the association between SHS exposure and depression symptoms among secondary school students in Malaysia by sociodemographic variables, smoking status and being victimized among participants in the NHMS 2017: AHS (N=27497)

*Variable*	*AOR*	*95% CI*
**Gender**		
Male ®	1	
Female	1.20	1.10–1.32
**Ethnicity**		
Malay ®	1	
Chinese	1.49	1.31–1.69
Indian	1.79	1.51–2.13
Bumiputra Sabah	1.34	1.13–1.59
Bumiputra Sarawak	0.88	0.73–1.06
Other	1.23	0.91–1.65
**Marital status of parents**		
Married ®	1	
Divorce/separated	1.23	1.11–1.37
**Physical attack at least once**		
Yes	1.37	1.25–1.51
No ®	1	
**Physical abuse at least once**		
Yes	1.46	1.28–1.65
No ®	1	
**Bullied at least one**		
Yes	1.84	1.64–2.06
No ®	1	
**Verbal abuse at least once**		
Yes	1.70	1.56–1.85
No ®	1	
**Having a close friend**		
Yes ®	1	
No	1.68	1.39–2.05
**Current smoker**		
Yes	1.51	1.33–1.71
No ®	1	
**Exposed to SHS**		
Yes	1.16	1.07–1.25
No ®	1	

® Reference categories. The sample was weighted based on the probability of selecting the school, classroom response rate of school level, class, and student, and poststratification adjustment on gender and form of the students.

## DISCUSSION

The study on the relationship between SHS and depression among a representative sample of secondary school students is novel. SHS exposure was associated with depression among secondary school students in Malaysia, after adjusting for the effect of socioeconomic status (gender, marital status of respondent parents/guardian), smoking status and being bullied, verbal and physical abuse, being physically attacked, and protective factors of having a close friend. The findings in the study were incongruent with those of Bandiera et al.^11^. In a nationally representative sample of US children and adolescents, secondhand smoke exposure was positively associated with symptoms of DSM-IV Major Depression Disorder throughout the entire cohort of non-smokers, even after controlling for age, sex, race/ethnicity, socioeconomic status, migraine, asthma, hay fever, maternal smoking during gestation, and allostatic load^11^. Similar findings were reported by Huang et al.^28^ among 3575 non-smokers, aged 15.0 ± 1.8 years; they reported a significant prevalence of probable depressive symptoms (OR=1.38; 95% CI: 1.16–1.63, for SHS in indoor public places; OR=1.24; 95% CI: 1.04–1.47, for SHS in homes; OR=1.57; 95% CI 1.29–1.92, for SHS in indoor campuses)18. SHS was also positively related to depression in the study of Lee and Kim^16^ among 51500 students in Korea. They found that the higher the level of exposure to SHS, the higher the prevalence of depression (odds of depression: 1.10-fold), with control variables such as age, gender, education level of parents, school achievement, economic status, residence, and drinking^16^. Similarly, Jacob et al.^14^ revealed that in their study, 37505 adolescents aged 12-15 years had never smoked using the standardized GSHS questionnaire. The rate of depressive symptoms rose from 23.0% in teenagers without exposure to secondhand smoke to 28.9% in those exposed to secondhand smoke daily in the preceding week after the adjustment for sex, age, food insecurity, and country. The difference in the level of odds between SHS exposure and depression, between this study and other studies might be due to the difference in frequency, intensity, and duration of SHS exposure among the respondents, in addition to the difference in the instruments or specific questions used to assess SHS and also depression/depression symptoms. Jacob et al.^14^ investigated the depressive symptoms among respondents by asking: ‘During the past 12 months, did you ever feel so sad or hopeless almost every day for two weeks or more in a row that you stopped doing your usual activities?’ (yes or no), while our study used the validated DASS 21 Questionnaire.

The results of this study complement existing research in the field on SHS and depression/depressive symptoms in teenagers from both low- and middle-income countries^14,15,28^, and in high-income countries^11,16^. First, the higher odds of depression and SHS exposure among adolescents may be explained by integrating biological and epidemiological aspects – the neurobiological process linked to SHS exposure in smokers and non-smokers. According to several animal studies, nicotine exposure affects the dopamine system both acutely and over time, which may cause long-term abnormalities in dopamine transport. Prolonged exposure to SHS lowers dopamine levels, which have also been linked to a higher risk of depression or low mood^9,10^. Second, a growing body of research indicates that frequent exposure to SHS may be a sign of a stressful work and home environment. Chronic stress of this kind has been linked to the worsening of depression symptoms by compromising neuroplasticity mechanisms and producing aberrant levels of neurotrophic factors^29^. For instance, structural alterations in the brain, such as the loss of dendritic spines and synapses, a reduction in dendritic arborization, and a decrease in glial cells in the hippocampus, are linked to long-term stress and major depression^30^. Third, exposure to secondhand smoke has been positively linked to several long-term medical disorders in children and adolescents (such as elevated asthma)^31^, some of which may predispose an individual to developing depression/depressive symptoms. Fourth, it has been demonstrated that physical pain (such as coughing and eye irritation) and SHS may raise perceived stress levels^32^. Stress is also a powerful predictor of depressive symptoms in adolescents, both male and female. Fifth, Certain studies indicate that prolonged exposure to secondhand smoke during stressful periods is associated with an increased risk of detrimental lifestyle and behavioral issues, including emotional and conduct disorders in youth. An unhealthy lifestyle manifests in lifestyle aspects such as physical exercise, eating habits, and sleep patterns, which can exacerbate mental health problems^33^.

The findings indicate that minimizing SHS exposure is crucial not only for preventing physical ailments, including ischemic heart disease, lung cancer, and asthma, but also for mitigating depressed symptoms in adolescents. Better implementation of reducing the SHS among adolescents in Malaysia is urgently needed, based on a plethora of research indicating that early-life exposure to tobacco smoke has detrimental consequences on children’s and adolescents’ mental health that last a lifetime^33^. Depression is estimated to occur among 1.1% of adolescents aged 10–14 years and 2.8% in those aged 15–19 years^34^. Because it results in a loss of functioning as a member of society, it places a significant financial burden on those who need treatment and their families and communities^35^. Reducing depression through monitoring and controlling secondhand smoke exposure may help to lower the number of suicide-related fatalities and injuries, as well as the associated financial costs.

The study’s findings necessitate regulations aimed at public venues commonly frequented by children and teenagers, such as schools, hospitals, and outdoor playgrounds, by amendment of smoke-free regulation by the Ministry of Health Malaysia. Moreover, in addition to safeguarding adolescents from secondhand smoke exposure, a more fundamental option to diminish secondhand smoke among teenagers is the decrease of the smoking population. Population-level strategies to reduce smoking encompass monitoring tobacco consumption and offering reasonable, accessible quitting support for smoking parents or guardians by enhancing the collaboration between the government and private health facilities to ensure more smokers can access quit-smoking clinics. Concerning SHS at home, educational initiatives for parents addressing the detrimental impacts of passive smoking on physical and mental health, are likely essential by mobilizing all the resources in primary health and community health volunteers to ensure the outreach of the information on the health hazards of smoking to all levels of the community. In addition, specific studies should focus on school students of Indian descent, given their higher exposure to SHS as found in this study, the pathway, and where they were exposed to SHS.

### Limitations

The study has several limitations. First, the cross-sectional study design only measures the association between SHS exposure and depression, whereas a longitudinal study is required to establish a causal or temporal relation between smoking and depression. Second, the data were obtained through self-report methods and SHS exposure was not quantified with detailed questionnaire data or biomarkers. It may result in information or recall bias; depression was measured using the DASS-21, which is not a clinical diagnostic measure, which is frequently used in research and practice to identify individuals with high distress who may be prone to develop psychopathologies. Thirdly, unmeasured confounding variables such as mental health of family members, alcohol consumption, the unknown source of SHS exposure (e.g. mother, father, or other), and illicit drug use, were not considered covariates in this study. Fourth, the data collected were in 2017; there may be some changes in the depression/depression symptoms of school students. In addition, we did not adjust for residual confounding, and the findings cannot be generalized to other populations (such as adolescents who did not attend secondary schools). Despite the limitations of cross-sectional studies, the current research utilized a substantial nationwide representative sample of Malaysian secondary school students, achieving a high response rate to the survey. Given the limitations, future studies should focus on longitudinal, intervention qualitative studies examining the extent and location of SHS exposure, primary sources, and exposure pathways in adolescent populations to enhance understanding of the causality and effectiveness of SHS prevention in mitigating depression risk. In addition, biochemical verification of SHS (e.g. salivary cotinine) should be emphasized, and clinical assessment of depressive symptoms is warranted. Furthermore, more variables associated with depression symptoms should be included in the study to ensure more valid results.

## CONCLUSIONS

To prevent and control school-going adolescents’ exposure to SHS, health education and smoking cessation among those who have close contact with adolescents should be enhanced. In addition, promoting more smoke-free areas, including houses and public places, should be intensified among secondary school-going adolescents in Malaysia as they transition to adulthood.

## Supplementary Material



## Data Availability

The data supporting this research are available from the authors on reasonable request.
