# Supplementary material for: Is secondhand smoke exposure associated with depressive symptoms among secondary school students in Malaysia? Findings from a national school-based study
Source: Tob Induc Dis. 2025 Mar 18;23:10.18332/tid/197278. doi: 10.18332/tid/197278 (PMC11915095; doi:10.18332/tid/197278)
Supplement: Supplementary file 1 [file TID-23-35-s1.pdf]

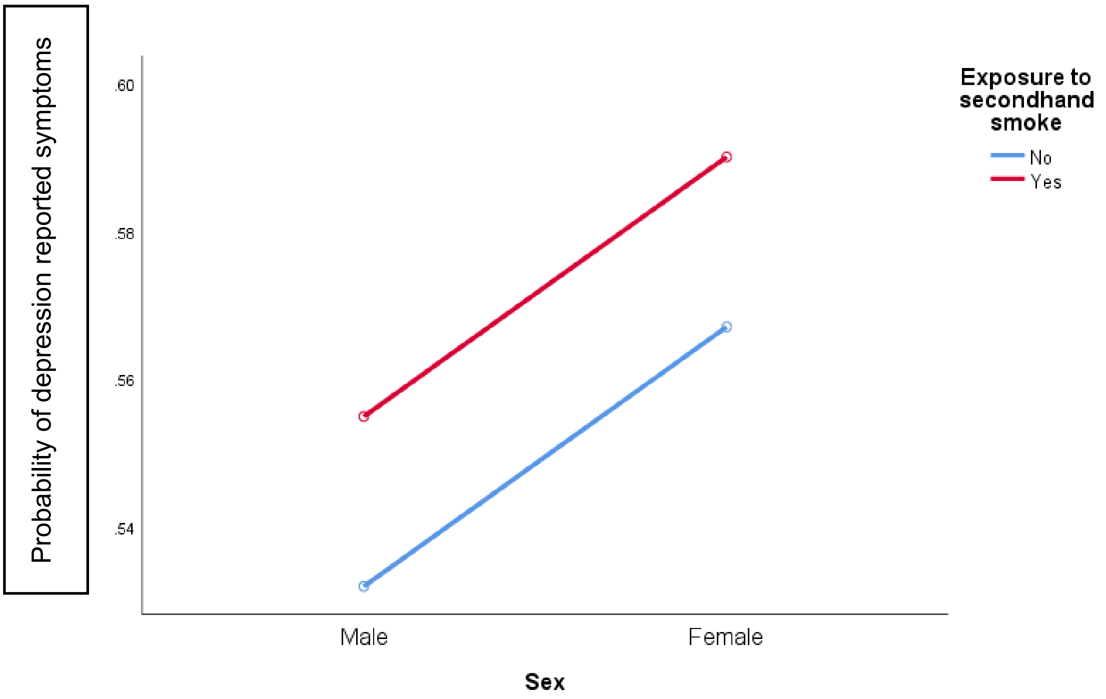

Figure 1 (a): Interaction between gender (sex) and second-hand smoke exposure

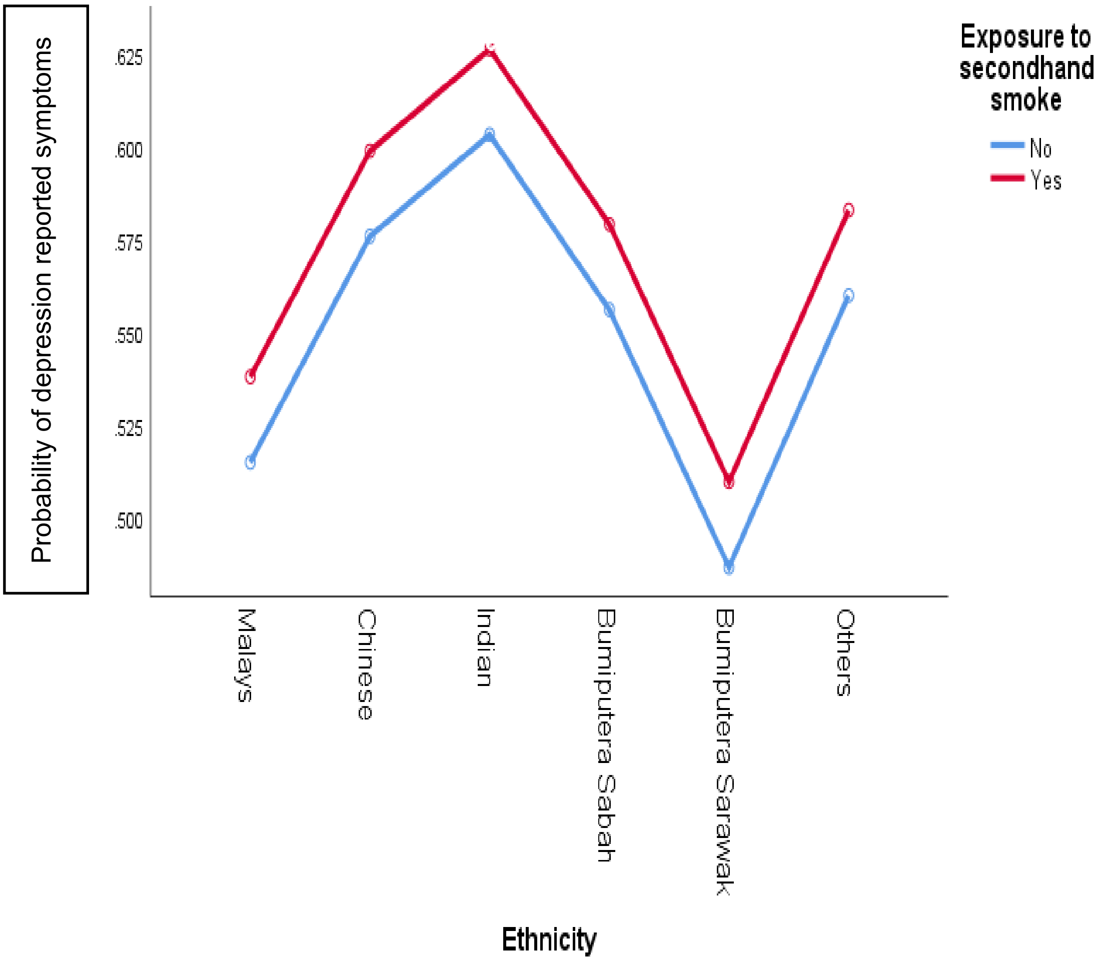

Figure 1 (b): Interaction between ethnicity and second-hand smoke exposure

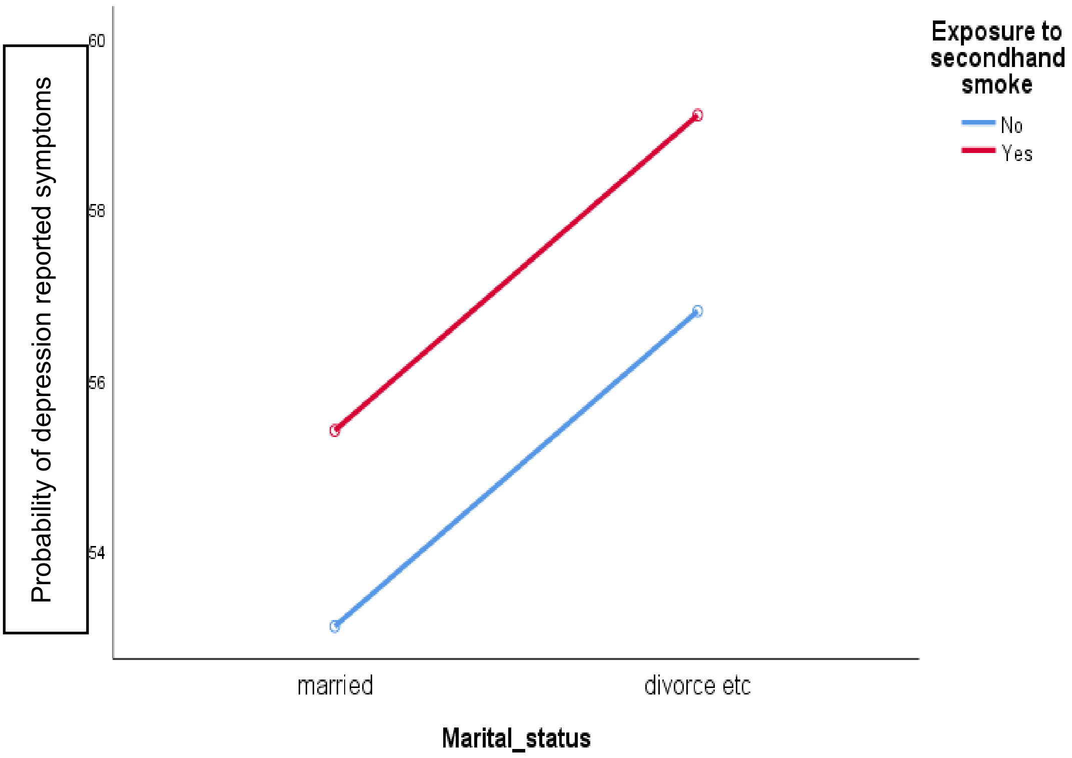

Figure 1 (c): Interaction between parental marital status and second-hand smoke exposure

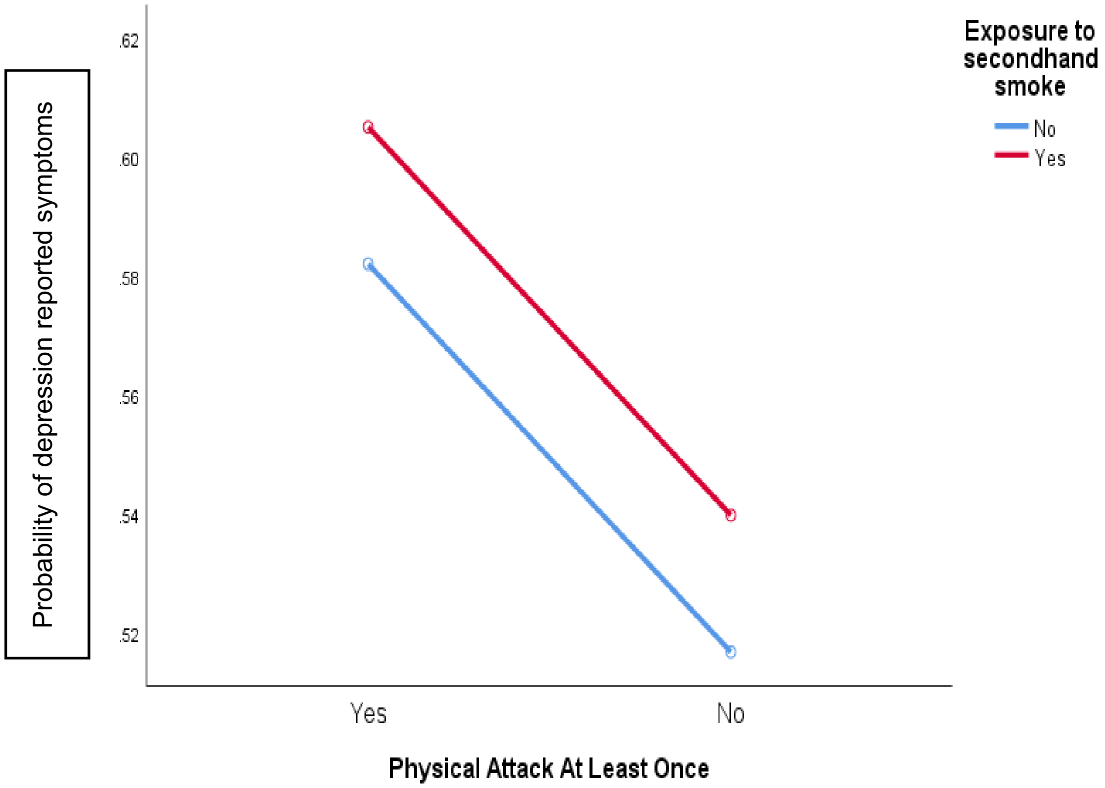

Figure 1 (d): Interaction between physical attack at least one and second-hand smoke exposure

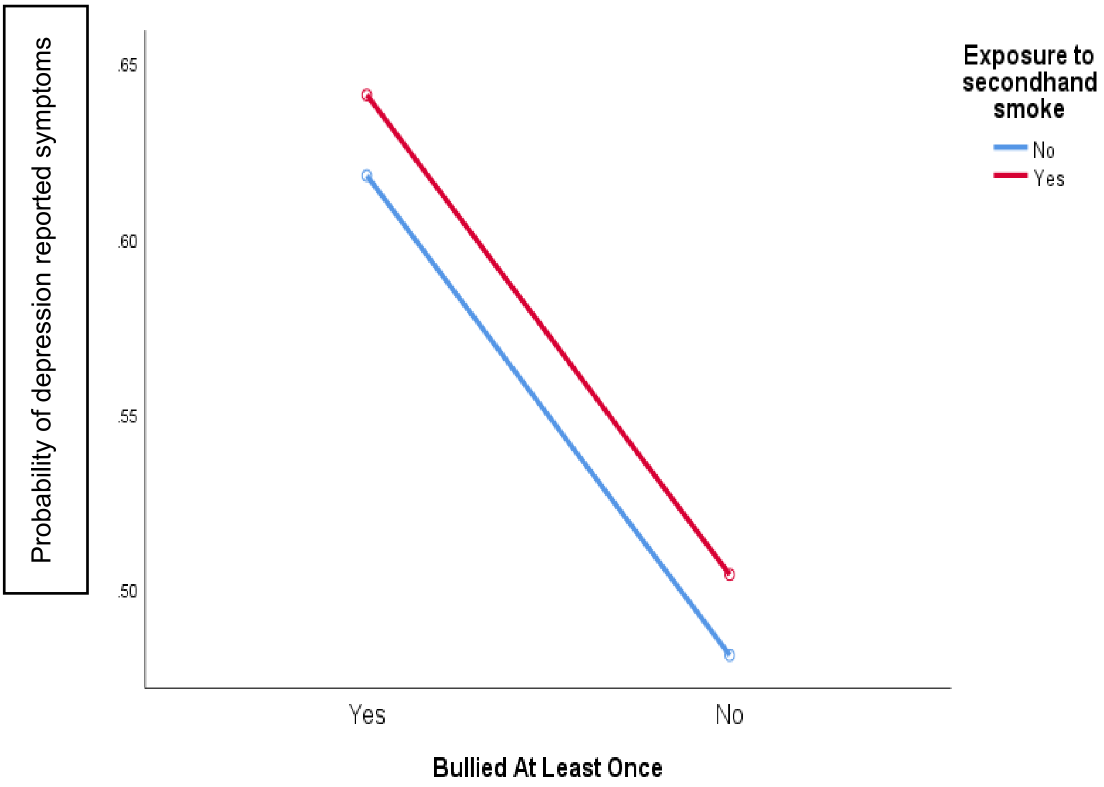

Figure 1 (e): Interaction between bullied at least once and second-hand smoke exposure

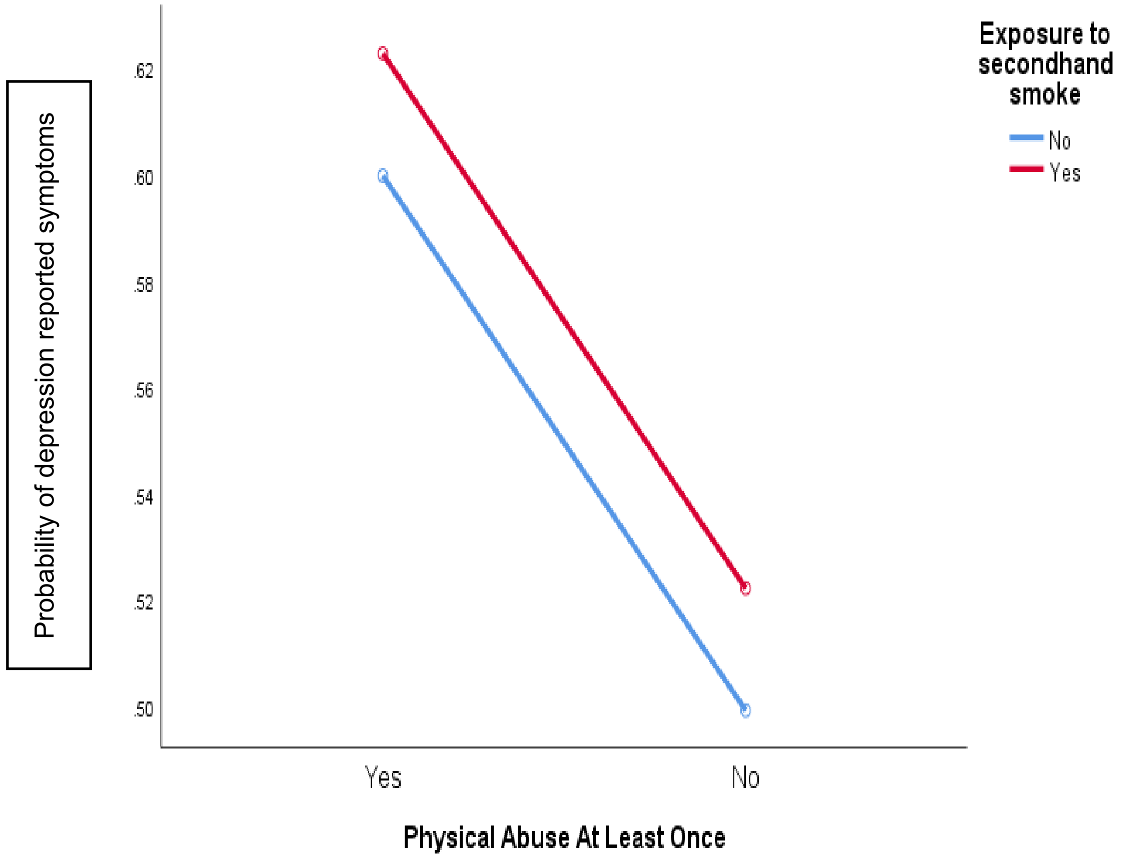

Figure 1 (f): Interaction between physical abuse at least once and second-hand smoke exposure

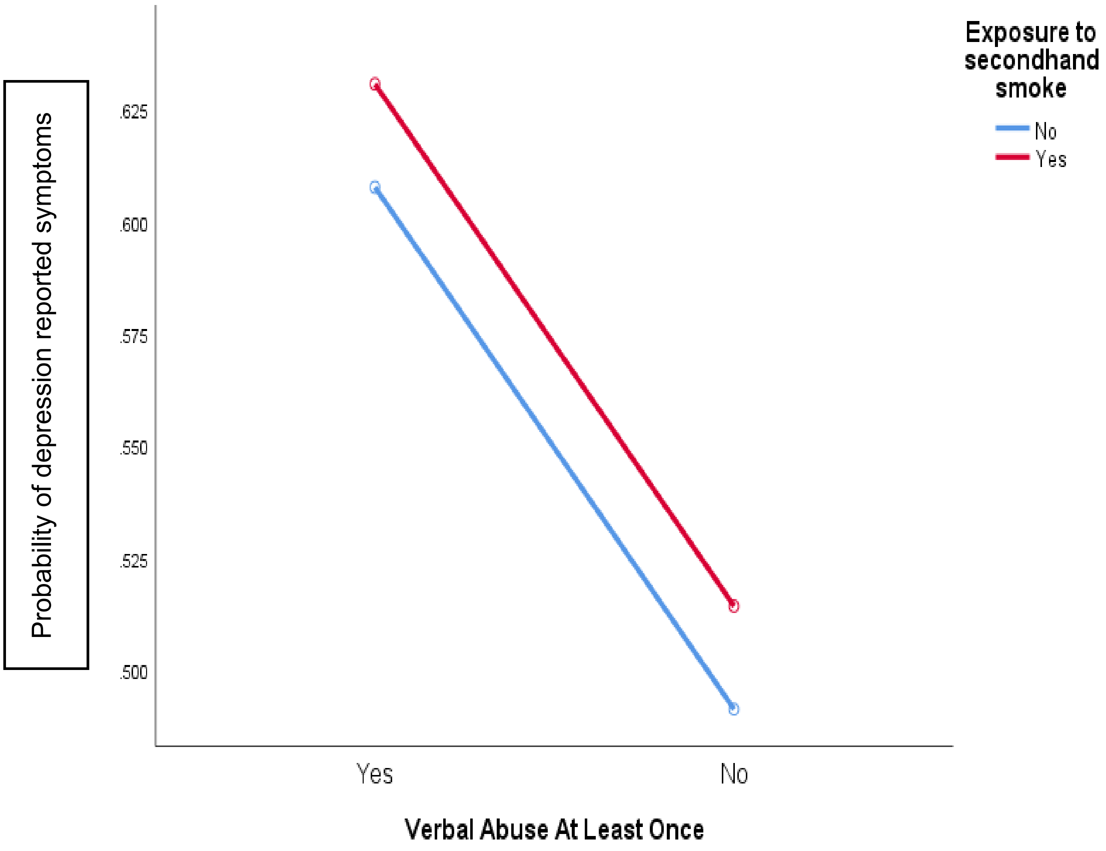

Figure 1 (g): Interaction between verbal abuse at least once and second-hand smoke exposure

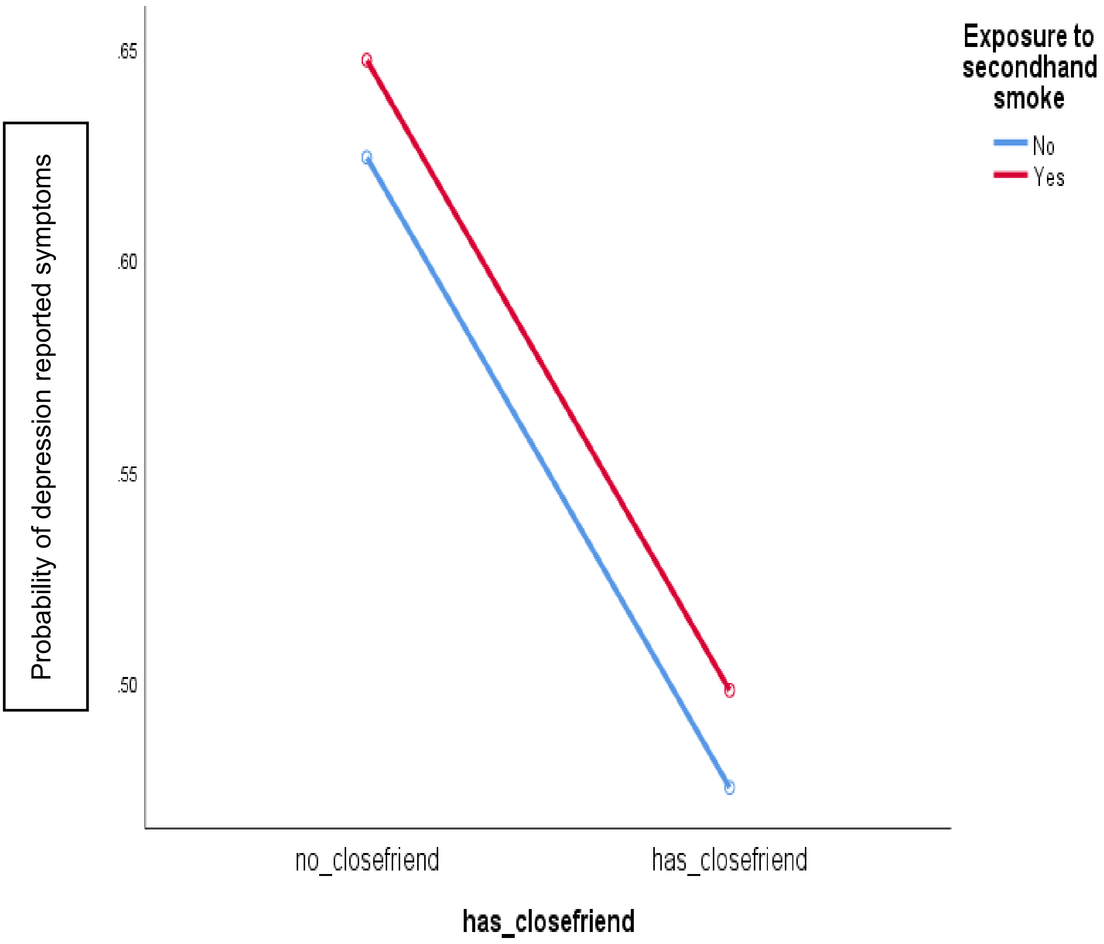

Figure 1 (h): Interaction between has close friend and second-hand smoke exposure

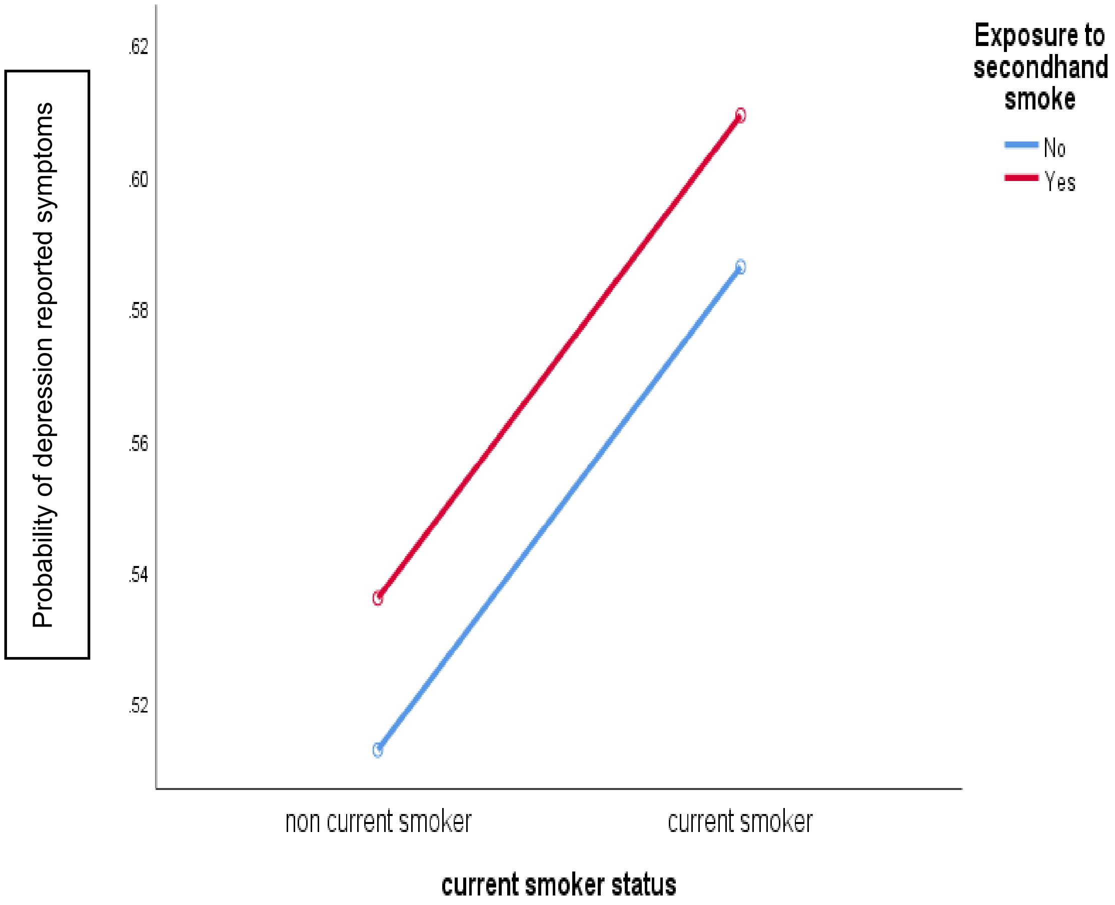

Figure 1 (i): Interaction between current smoking status and second-hand smoke exposure

© 2025 Lim K.H. et al.
